# Functional coronary vascular disease: endotype-based classification, diagnosis, and targeted management

**DOI:** 10.3389/fcvm.2026.1811419

**Published:** 2026-07-20

**Authors:** Omar El Khatib, Ashraf Al Azzoni, Ronney Shantouf, Bassam Atallah

**Affiliations:** 1 Department of Pharmacy Services, Cleveland Clinic Abu Dhabi, Al Maryah Island, Abu Dhabi, United Arab Emirates; 2 Cleveland Clinic Abu Dhabi, Heart and Vascular Institute, Al Maryah Island, Abu Dhabi, United Arab Emirates; 3 College of Pharmacy, Dubai Medical University, Dubai, United Arab Emirates

**Keywords:** coronary (artery) spasm, coronary flow reserve, microvascular dysfunction, vasospam, vasospastic angina

## Abstract

**Background:**

Angina and exertional dyspnea are hallmark manifestations of coronary artery disease (CAD), traditionally linked to obstructive epicardial lesions. However, a substantial subset of patients experience angina despite angiographically non-obstructive coronary arteries (<50%), termed angina with non-obstructive coronary arteries (ANOCA). ANOCA may progress to ischemia with non-obstructive coronary arteries (INOCA), myocardial infarction with non-obstructive coronary arteries (MINOCA), and ischemic cardiomyopathy. Despite advances in diagnostic modalities, the underlying pathophysiology remains underappreciated, and management often lacks precision.

**Pathophysiology:**

ANOCA and INOCA encompass heterogeneous functional and structural coronary abnormalities, including endothelial dysfunction, impaired vasodilation, and inappropriate vasoconstriction of epicardial or microvascular vessels. Epicardial arteries serve primarily as conduits, whereas the microcirculation regulates coronary flow, mediating myocardial perfusion through nitric oxide, adenosine, and prostacyclin signalling. Disruption of these mechanisms precipitates ischemia despite normal epicardial anatomy, defining distinct endotypes with diagnostic and therapeutic relevance.

**Diagnosis and management:**

Evaluation involves stepwise assessment from non-invasive testing, including ECG, laboratory studies, echocardiography, and coronary computed tomography, to invasive functional testing for endotype characterization. Management should integrate endotype-specific pharmacologic therapies such as calcium channel blockers, nitrates, ranolazine, ACE inhibitors, and beta-blockers with non-pharmacologic interventions including lifestyle modification and overall cardiovascular risk reduction.

**Conclusion:**

Personalized, endotype-guided strategies targeting both pathophysiology and modifiable risk factors are essential to optimize symptom control, prevent disease progression, and reduce major adverse cardiovascular events in patients with ANOCA and INOCA.

## Introduction

## 1 Background

Angina and exertional dyspnea are hallmark symptoms of coronary artery disease (CAD), traditionally attributed to obstructive lesions within the epicardial coronary arteries. While obstructive CAD remains the most thoroughly characterized subtype, there is growing recognition of a substantial subset of patients who experience angina despite the absence of angiographically significant stenoses (<50%), referred to as angina with non-obstructive coronary arteries (ANOCA). Recent data suggest that ANOCA may be in fact more prevalent than previously appreciated and may represent the predominant clinical phenotype (1). ANOCA may evolve into more severe phenotypes such as ischemia with non-obstructive coronary arteries (INOCA), myocardial infarction with non-obstructive coronary arteries (MINOCA), and ultimately, ischemic cardiomyopathy (ICM). Although diagnostic and therapeutic advances have improved our ability to identify and categorize these entities, the underlying pathophysiologic mechanisms are often underexplored and insufficiently targeted in current management strategies, leaving many patients without effective, individualized treatment (1, 2).

## 2 Epidemiology and prognosis

Emerging data indicate that as many as 70% of patients referred for elective coronary angiography due to anginal symptoms do not exhibit obstructive coronary disease, instead meeting criteria for ANOCA (3, 4). Importantly, the prognosis in ANOCA and INOCA is closely tied to early recognition and the implementation of targeted, mechanism-specific therapies (4). The growing clinical burden of ANOCA, INOCA, and MINOCA calls for the development and implementation of a structured, endotype-driven diagnostic algorithm. Such an approach would facilitate tailored therapeutic interventions and enable more standardized follow-up pathways. Patients with INOCA who have non-obstructive coronary atheroma are at increased risk of cardiovascular events and should receive intensive therapy directed towards major adverse cardiac event (MACE) risk reduction (5).

## 3 Pathophysiology

The pathophysiologic landscape of ANOCA and INOCA is markedly heterogeneous, encompassing a broad spectrum of functional and structural perturbations within the coronary circulation. Although the epicardial coronary arteries function predominantly as passive conduits for myocardial perfusion, they are inherently limited in their ability to modulate coronary blood flow in response to metabolic demand (4, 6). By contrast, the coronary microvasculature is the chief determinant of coronary blood flow (CBF), orchestrating perfusion through tightly regulated alterations in arteriolar tone. This process is governed by vasodilatory signals including cardiomyocyte-derived adenosine during ischemia, as well as nitric oxide (NO) and prostacyclins released from the endothelium which collectively augment coronary flow reserve (CFR) (7, 8). Disruption of this intricately coordinated system -whether through endothelial dysfunction, microvascular remodeling, or impaired vasodilatory signaling- may precipitate myocardial ischemia despite angiographically normal epicardial vessels. These disturbances can be delineated into distinct mechanistic endotypes with meaningful pathophysiologic, diagnostic, and therapeutic relevance (3).

## 4 Clinical presentation and diagnosis

Multiple studies have documented the sex-biased nature of ANOCA, with the disease being far more prevalent in females (9, 10). Symptom presentation in suspected chronic coronary syndromes (CCS) varies markedly across individuals, with demographic and clinical characteristics–including age, sex, and race–modulating the manifestation and interpretation of chest pain (11). Consequently, a systematic, multimodal assessment is essential prior to considering invasive coronary testing.

The initial evaluation should comprise a 12-lead ECG, targeted laboratory studies to exclude alternative non-cardiac drivers of ischemia –including anemia, thyroid dysfunction, or other metabolic disturbances– and a review of cardiovascular risk profiles. Resting echocardiography is similarly recommended to identify structural or functional cardiac abnormalities that could account for chest pain. Further testing should be informed by the estimated clinical likelihood of CCS, integrating standardized tools such as the Risk Factor–weighted Clinical Likelihood (RF-CL) model and coronary artery calcium scoring (CACS) (12), the latter offering a high negative predictive value (NPV) for obstructive CAD (13). Advanced diagnostic modalities should then be reserved for those patients who, on the basis of this structured workup, meet criteria for higher-risk classification (14).

To promote consistency in the diagnosis of vasospastic angina, the Coronary Vasomotion Disorders International Study Group (COVADIS) has established formal diagnostic criteria for functional coronary artery disease (15). In patients for whom clinical suspicion remains high, further evaluation should include exercise electrocardiography or, preferably, coronary computed tomography angiography (CCTA), the latter having been associated with superior patient outcomes (16). Exercise ECG stress test utility to diagnose obstructive CAD is limited due to its poor sensitivity and specificity (17). Its diagnostic performance is particularly limited in patients exhibiting resting ECG abnormalities from concurrent pathologies such as left bundle branch block. Despite these constraints, exercise ECG maintains a role in stratifying cardiovascular risk.

### 4.1 Anatomical imaging: CCTA

Assessment of coronary anatomy can be done with visual characterization of epicardial disease and be paired with physiologic evaluation of suspected stenoses. Coronary computed tomography angiography (CCTA) enables high-resolution anatomical imaging of the epicardial coronary tree, and obstructive lesions have traditionally been defined using visual thresholds of approximately 70% diameter reduction. Nevertheless, growing evidence indicates that lesions outside these angiographic thresholds may still exert meaningful functional and hemodynamic impact (18). Consequently, complementing CCTA with non-invasive fractional flow reserve estimation through FFR-CT serves as an effective pre-screening tool before invasive angiography, reducing the likelihood of unnecessary testing (19). In patients with symptoms consistent with myocardial ischemia but non-obstructive epicardial findings on CCTA, microvascular etiology and coronary artery spasm should be strongly considered.

Additional non-invasive testing may include myocardial perfusion scintigraphy using single-photon emission computed tomography (SPECT), which assists in identifying obstructive coronary artery disease, as well as stress echocardiography performed with or without intravenous ultrasound contrast agents to evaluate myocardial ischemia through detection of stress-induced regional systolic wall-thickening abnormalities. However, stress echocardiography should be regarded as a complementary tool within the broader clinical assessment rather than as a definitive method to exclude CCS given limited sensitivity. Stress echocardiography may substantially under-detect ischemia in patients with microvascular dysfunction that does not extend to the subendocardium, as is often the case in CCS (20). Additionally, cardiac magnetic resonance (CMR) with quantitative perfusion mapping has been validated against invasive coronary physiology and can identify CMD (21).

Invasive coronary functional testing enables direct evaluation of both epicardial and microvascular circulatory function through sequential measurement of intracoronary pressures and flow indices in response to pharmacologic provocation (3, 22). This technique offers greater diagnostic certainty and is associated with improved clinical outcomes (23). Thermodilution-derived measurements obtained during invasive testing allow quantification of epicardial stenosis severity through fractional flow reserve assessment.

Coronary microvascular dysfunction is diagnosed when a reduction in coronary flow reserve (CFR) is accompanied by elevated indices of microvascular resistance, including IMR, HMR, or MRR, in the absence of angiographically significant epicardial disease (24). CFR is obtained using a coronary wire incorporating both Doppler and pressure sensors to measure averaged peak flow velocity (APV) during resting and hyperaemic states as a surrogate index of coronary blood flow (25). COVADIS has outlined explicit indications for transitioning to invasive coronary functional testing following initial diagnostic workup (15).

Because microvascular dysfunction reflects a broad and multifaceted pathophysiologic continuum rather than a uniform disease state, accurate diagnostic delineation is crucial for selecting mechanism-specific therapeutic strategies (3, 26). In patients who exhibit significant anginal symptoms but maintain an FFR above 0.8, further pathophysiological investigation is indicated. We will be delineating the currently recognized endotypes, corresponding targeted endotype-specific therapies, and universal disease-modifying therapies.

## 5 Precision endotyping

ANOCA and INOCA encompass a spectrum of abnormal coronary responses that include impaired vasodilatation, either through endothelium dependent or independent mechanisms, and inappropriate vasoconstriction involving the epicardial arteries or the microcirculation. These pathophysiologic patterns define separate endotypes that require targeted diagnostic assessment and tailored therapy. Subsequent sections review the diagnostic evaluation for each endotype, with Table 1 providing an overview of key diagnostic indices and corresponding management options.

**Table 1:** Diagnostic approaches, pharmacological interventions, and risk-reduction strategies across chronic coronary syndrome endotypes.

Endotype	Diagnostic evidence on ICA	Pharmacological therapy	Risk reduction measures
Coronary Microvascular dysfunction	Positive response to low-dose intracoronary Ach with vasoconstriction instead of expected vasodilation; CFR <2, IMR >25, HMR >2.5 mmHg/cm/s	Beta blockers CCBs Ranolazine Coronary sinus reducer	Statins Antiplatelets ACEI Optimal BP control Optimal glycemic control Smoking Cessation Weight management Promoting exercise
Impaired vasodilation (endothelium-independent)	Preserved endothelial function on Ach provocation testing; reduced endothelium-independent vasodilation with intracoronary adenosine; CFR <2, IMR <25	Beta blockers CCBs	
Epicardial vasospastic angina	≥90% transient angiographic narrowing during provocation with acetylcholine, with ischemic symptoms and ECG changes	CCBs Long-acting nitrates Nicorandil Sympathectomy or spinal cord stimulation	
Microvascular vasospastic angina	<90% narrowing on ICA with acetylcholine provocation; ischemic symptoms and ECG changes		
Mixed endotypes	Evidence of more than one pathophysiologic mechanism on ICA	Management per involved endotypes	

ICA, invasive coronary angiography; Ach, acetylcholine; CFR, coronary flow reserve; IMR, index of microcirculatory resistance; ACEi, angiotensin-converting enzyme inhibitor; CCBs, calcium channel blockers; BP, blood pressure; ECG, electrocardiogram.

### 5.1 Coronary physiology testing

In individuals with intact endothelial function, administration of low dose acetylcholine (Ach) induces the release of endothelium-dependent relaxation factors, predominantly nitric oxide, through binding to muscarinic receptors on the endothelium (27).

When endothelial function is impaired, Ach produces vasoconstriction by acting directly on muscarinic receptors on vascular smooth muscle, as the dysfunctional endothelium cannot generate sufficient NO to oppose this effect (3). In order to diagnose vasospastic angina, intra-coronary Ach testing is needed. Due to its short half-life, Ach must be administered intracoronarily (28). Although multiple protocols exist for acetylcholine dosing, all follow the same principle of incremental administration with a subsequent bolus, up to a maximum of 200 micrograms, to induce vasospasm (3, 23, 28, 29). The vasoconstrictive effects of Ach are short-lived and can be reversed with 200 micrograms of intracoronary nitroglycerine if necessary (3). The test is considered positive for epicardial spasm if angina, ischemic ECG changes, and ≥90% angiographic narrowing are observed. If only angina and ischemic ECG changes elicited with a lumen reduction of less than 90% then this indicates microvascular vasospastic dysfunction (3). Patients with coronary microvascular dysfunction typically show a coronary flow reserve (CFR) below 2 and an index of microvascular resistance (IMR) above 25 (30).

### 5.2 Coronary microvascular dysfunction and impaired vasodilation (endothelium-independent)

Healthy endothelium regulates vascular tone through the release of vasoactive substances, including nitric oxide (NO), endothelin, prostacyclin, and angiotensinogen, which are essential for maintaining adequate coronary blood flow during periods of increased metabolic demand (31, 32). Endothelial dysfunction arises early in atherogenesis and diminishes the normal vasodilatory response to stress (32). Endothelium-dependent CMD can be attributed to reduced production or action of these relaxing mediators (33).

Some patients may have preserved endothelial function but exhibit impaired vasodilation at the level of vascular smooth muscle, which limits maximal coronary dilation under stress. Because vascular smooth muscle cells constitute the final common pathway for vasorelaxation, impairment at this level can also attenuate endothelium-dependent responses. Assessment of endothelium-independent vasodilation is achieved using intracoronary adenosine, a hyperemic agent that directly relaxes smooth muscle via A2A receptor activation (34).

#### 5.2.1 Beta blockers (BBs)

The therapeutic benefit of beta-blockers in endothelium-independent microvascular angina arises from their ability to reduce myocardial oxygen demand by decreasing contractility and to improve oxygen supply through prolongation of diastolic perfusion time (35). Caution is required in vasospastic angina, as beta-blockers lacking alpha-blocking properties may worsen vasospasm due to unopposed alpha-mediated constriction in absence of *β*2-mediated vasodilation (25, 35, 36). Accordingly, beta-blockers are recommended as first-line therapy for endothelium-independent microvascular angina but are generally avoided in patients with vasospastic angina, consistent with current American and European guidelines (3, 30).

#### 5.2.2 Angiotensin-converting enzyme inhibitors (ACEi)

ACEi promote coronary microvascular vasodilation by inhibiting angiotensin II synthesis and increasing local bradykinin levels, which in turn stimulates endothelial release of nitric oxide (37). These mechanisms render ACEi particularly beneficial for patients with coronary microvascular angina driven by endothelial dysfunction. This therapeutic effect was demonstrated in the WISE trial, in which quinapril significantly improved coronary flow reserve and reduced anginal symptoms compared with baseline (38). Based on these findings, ACE inhibitors are recommended in patients presenting with microvascular angina secondary to endothelial dysfunction (3, 30).

#### 5.2.3 Ranolazine

Ranolazine is a late sodium current inhibitor in cardiac myocytes, which reduces intracellular sodium and, consequently, calcium influx via sodium-calcium exchangers. This mechanism decreases myocardial oxygen demand by lowering wall tension (39). The efficacy of ranolazine in stable angina has been demonstrated in the CARISA and ERICA trials, where patients randomized to ranolazine exhibited increased exercise tolerance and a reduced frequency of anginal episodes (40, 41).

#### 5.2.4 Coronary Sinus reducer (CSR)

For patients with refractory angina, implantation of a coronary sinus reducer (CSR) offers an additional treatment option for patients with refractory angina pectoris (RAP). The device, positioned across the coronary sinus valve, elevates coronary venous pressure, leading to vessel dilation, reduced microvascular resistance, and redistribution of blood flow to ischemic myocardial regions (42). The clinical efficacy of the CSR is well-established in the context of obstructive CAD, where it significantly reduces anginal frequency and improves quality of life for patients who are poor candidates for conventional revascularization (43). Increasingly, its utility is expanding into non-obstructive CAD; a recent phase II trial demonstrated that the CS Reducer is associated with significant improvement in angina, quality of life, and coronary microvascular function in patients with CMD (44). Nevertheless, the definitive role of CSR in RAP secondary to non-obstructive CAD remains an evolving landscape, with ongoing clinical trials actively underway to further delineate its precise position within the therapeutic hierarchy (45, 46). On basis of this evidence, CSR implantation is graded a Class IIb recommendation in ESC guidelines (3).

### 5.3 Vasospastic angina

#### 5.3.1 Epicardial vasospastic angina (VSA)

This endotype is defined by vasospasm of one or more epicardial coronary arteries in the absence of significant stenosis. The underlying pathophysiology is complex and multifactorial, with a key role for vascular smooth muscle hyperreactivity mediated by RhoA/Rho-kinase activation, which enhances calcium sensitization (47). In addition, several vasoactive mediators, including serotonin and histamine, may contribute to the development of vasospasm (48, 49). Patients with epicardial vasospastic angina were also found to exhibit higher serum levels of high-sensitivity C-reactive protein (hsCRP), suggesting that inflammation may influence disease pathophysiology, potentially by disrupting endothelial NO synthesis (50).

#### 5.3.2 Microvascular vasospastic angina (MVA)

The coronary microvasculature, which constitutes the vast majority of the myocardial circulation, plays a central role in regulating coronary resistance and blood flow (25). MVA is characterized by dynamic vasoconstriction of the coronary microcirculation, resulting in transient myocardial ischemia in the absence of epicardial coronary artery spasm (51). This distinct endotype of coronary microvascular dysfunction is driven by enhanced microvascular vasomotor reactivity and has important therapeutic implications, as patients often derive greater benefit from vasodilator-based strategies, particularly calcium channel blockers.

##### 5.3.2.1 Calcium channel blockers (CCBs)

Building on the pathophysiology in which calcium influx into vascular smooth muscle cells plays a central role in epicardial vasospastic angina, calcium channel blockers are considered first-line therapy to prevent anginal episodes and related complications (3). By inhibiting voltage-dependent L-type calcium channels, these agents reduce coronary vasoreactivity and smooth muscle contraction (52). Clinical evidence supports the use of both dihydropyridine and non-dihydropyridine calcium channel blockers, individually or in combination, with demonstrated efficacy and safety across multiple studies (53–55).

##### 5.3.2.2 Nitrates

Nitrates of varying half-lives act by releasing nitric oxide, which increases cyclic GMP in vascular smooth muscle cells, ultimately resulting in coronary vasodilation. In addition, venodilation induced by nitrates reduces preload, thereby decreasing myocardial oxygen demand (56, 57). However, evidence from the VA-KOREA study demonstrated that long-acting nitrate regimens were associated with an increased two-year incidence of acute coronary syndromes (ACS) (58). Similarly, an observational study of 1,154 patients found that nitrate-based therapies were linked to a higher risk of MACE (59). Several mechanisms have been proposed to explain these findings, including the development of nitrate tolerance with prolonged use, which may limit long-term efficacy (60), and rebound vasoconstriction during nitrate-free intervals (61). Short-acting sublingual nitroglycerine remains the preferred option for acute symptomatic relief (3).

##### 5.3.2.3 Nicorandil

Nicorandil mediates its anti-anginal effects through a dual mechanism. By donating nitric oxide, it increases cyclic GMP levels, leading to vasodilation, while activation of ATP-sensitive potassium channels (KATP) induces membrane hyperpolarization, closure of voltage-dependent calcium channels, and reduced calcium entry into vascular smooth muscle cells. Together, these mechanisms result in rapid coronary vasodilation (62). According to the 2024 ESC guidelines, nicorandil carries a Class IIb recommendation as an adjunctive therapy for patients with vasospastic angina who do not achieve adequate symptom relief with first line therapy, CCBs (3). Evidence to this approach generally stems from multiple randomized controlled trials (62–64).

##### 5.3.2.4 Trimetazidine

Trimetazidine exerts its anti-anginal effects by modulating myocardial energy metabolism to reduce oxygen demand. Inhibition of the enzyme long-chain 3-ketoacyl coenzyme A thiolase (LC 3-KAT) shifts myocardial substrate utilization toward glucose oxidation and suppresses energy-intensive fatty acid metabolism, resulting in decreased oxygen consumption during ischemic episodes (65). Accordingly and based on the published observational studies, the 2024 ESC guidelines recommend trimetazidine as an adjunct to standard therapy, assigning it a Class IIb recommendation (3).

### 5.4 Non-pharmacological therapies

For patients who remain symptomatic despite maximal medical therapy, there are surgical options including sympathectomy as well as spinal cord stimulation (SCS). SCS is endorsed by the ESC guidelines as a therapeutic option of last resort (25). The mechanism of SCS involves modulation of sympathetic activity, leading to improved myocardial blood flow, as well as attenuation of anginal pain transmission (66, 67). Evidence from a meta-analysis of 12 randomized trials by Pan and colleagues indicates that SCS significantly reduces the frequency of angina episodes in patients with persistent symptoms (68).

### 5.5 Endotype combinations

In individuals with multiple endotypes confirmed on invasive coronary evaluation, therapy should target each affected pathway. Pharmacologic treatment should be integrated with lifestyle interventions, including smoking cessation, management of body weight, and avoidance of environmental and social triggers such as cold exposure, to provide a comprehensive risk reduction approach (3).

## 6 Universal disease-modifying therapies

Individuals with ANOCA who exhibit non-obstructive atheromas are at an increased risk for both disease progression and MACE (25, 69) warranting intensive risk-reduction measures. These strategies encompass rigorous blood pressure management to limit microvascular remodeling, lifestyle interventions, and the initiation and careful titration of anti-anginal therapy targeted to the patient's specific endotype. Achieving optimal glycemic control in patients with diabetes is critical to prevent endothelial dysfunction and the development of diabetic cardiomyopathy. Interestingly, metformin has been demonstrated in a small randomized controlled trial to improve coronary blood flow during acetylcholine provocation, an effect likely mediated by its capacity to reduce insulin resistance (70).

Statin therapy should be initiated in all patients with chronic coronary syndromes, irrespective of endotype, with a goal of reducing low-density lipoprotein cholesterol (LDL-C) by at least 50 percent from baseline (3). The cardioprotective effects of statins extend beyond LDL-C lowering, as their pleiotropic properties reduce vascular inflammation and improve endothelial function (71).

### 6.1 Antiplatelets

For patients with chronic coronary syndrome and a history of myocardial infarction or percutaneous coronary intervention, the 2024 ESC guidelines firmly endorse low-dose aspirin (ASA) therapy as a Class I recommendation for secondary prevention (3). Conversely, the therapeutic role of antiplatelet agents in vasospastic angina without obstructive coronary artery disease (CAD) remains poorly defined, with current observational data consistently questioning its utility. A systematic review of observational studies demonstrated that ASA administration yielded no significant reduction in major adverse cardiovascular event (MACE) rates within this cohort (72). Furthermore, Lim and colleagues reported that ASA use was paradoxically associated with an elevated MACE risk among patients with positive spasm provocation testing and non-significant coronary artery stenosis (73). The hypothesized mechanism driving this adverse outcome is the potential exacerbation of coronary vasoconstriction secondary to prostacyclin inhibition.

## 7 Future directions

ANOCA remains a rapidly evolving field in which current knowledge likely represents only a fraction of the underlying pathophysiology. In a recent observational study of 1,001 patients without angiographically significant epicardial stenoses at baseline (FFR >0.80), invasive evaluation of coronary vasomotor function delineated eight distinct ANOCA endotypes, each potentially amenable to mechanism-guided therapeutic strategies (74). These findings underscore the need for future investigations to further elucidate these mechanistic pathways and to refine targeted treatment approaches that may improve clinical outcomes.

Moreover, numerous genetic variants have been identified as potential theragnostic biomarkers, including the rs9349379 minor G allele which inhances endothelin-1 expression, thereby promoting vasoconstriction. The ongoing PRIZE trial will assess the impact of the Endothelin-A receptor antagonist, Zibotentan, in combination as adjunctive therapy in patients with microvascular angina (75). Additionally, *PHACTR1* and *ATP2B1* have been identified as potential mediators of CMD, as they are involved in NO signalling, consequently may serve as potential therapeutic targets in the future (9).

Sodium-glucose cotransporter-2 inhibitors have demonstrated improvement in coronary microvascular function in diabetic patients (76). The potential role of sodium-glucose cotransporter-2 inhibitors in women with ANOCA is being studied in the SMILE trial (Effect of Dapagliflozin on Microvascular Function in Women With Symptoms of Coronary Artery Disease; NCT05762952).

Furthermore, inflammation has been demonstrated to be implicated in the pathophysiology of ANOCA (9). Investigation by Schroder and colleagues demonstrated multiple biomarkers involved in the IL-6 proinflammatory pathways to be markedly elevated in patients with ANOCA (77). Randomized trials assessing whether the interleukin-6 receptor antagonist tocilizumab has a role in disease management are anticipated.

## 8 Conclusion

**Figure 1:**
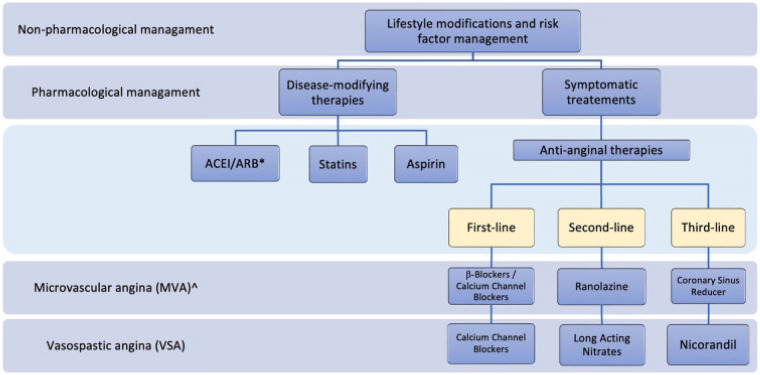
Schematic overview of chronic coronary syndrome endotypes and the evidence-based strategies employed in management.

CCS have evolved into a recognized spectrum of heterogeneous endotypes, reflecting the complexity of coronary pathophysiology. Because clinical presentations often overlap with those of coronary artery disease, accurate identification of individual endotypes is crucial. Optimal management requires a comprehensive, personalized approach that combines targeted pharmacologic therapies with non-pharmacologic strategies, including lifestyle interventions and cardiovascular risk factor control, such as that depicted in Figure 1. Such an approach is essential to alleviate symptoms, prevent disease progression, and reduce the incidence of major adverse cardiovascular events.
